# Extracellular Membrane Vesicles from Lactobacilli Dampen IFN-γ Responses in a Monocyte-Dependent Manner

**DOI:** 10.1038/s41598-019-53576-6

**Published:** 2019-11-19

**Authors:** Manuel Mata Forsberg, Sophia Björkander, Yanhong Pang, Ludwig Lundqvist, Mama Ndi, Martin Ott, Irene Buesa Escribá, Marie-Charlotte Jaeger, Stefan Roos, Eva Sverremark-Ekström

**Affiliations:** 1 The Department of Molecular Biosciences, The Wenner-Gren Institute, Stockholm University, Stockholm, Sweden; 2 The Department of Molecular Sciences, Swedish University of Agricultural Sciences, Uppsala, Sweden; 3 The Department of Biochemistry and Biophysics, Stockholm University, Stockholm, Sweden

**Keywords:** Cytokines, Bacteria

## Abstract

Secreted factors derived from *Lactobacillus* are able to dampen pro-inflammatory cytokine responses. Still, the nature of these components and the underlying mechanisms remain elusive. Here, we aimed to identify the components and the mechanism involved in the *Lactobacillus*-mediated modulation of immune cell activation. PBMC were stimulated in the presence of the cell free supernatants (CFS) of cultured *Lactobacillus rhamnosus* GG and *Lactobacillus reuteri* DSM 17938, followed by evaluation of cytokine responses. We show that lactobacilli-CFS effectively dampen induced IFN-γ and IL-17A responses from T- and NK cells in a monocyte dependent manner by a soluble factor. A proteomic array analysis highlighted *Lactobacillus*-induced IL-1 receptor antagonist (ra) as a potential candidate responsible for the IFN-γ dampening activity. Indeed, addition of recombinant IL-1ra to stimulated PBMC resulted in reduced IFN-γ production. Further characterization of the lactobacilli-CFS revealed the presence of extracellular membrane vesicles with a similar immune regulatory activity to that observed with the lactobacilli-CFS. In conclusion, we have shown that lactobacilli produce extracellular MVs, which are able to dampen pro-inflammatory cytokine responses in a monocyte-dependent manner.

## Introduction

The influence of gut commensal bacteria on immune development and function is well established^1,2^. Still, the relationship between the host and gut bacteria is complex and although microbial diversity is an important factor in maintaining immune homeostasis^3–5^, specific bacterial organisms such as lactobacilli have been found to have key roles in shaping immune cell composition and function locally as well as systemically^6–9^.

Probiotic bacteria are defined as “live microorganisms that, when administered in adequate amounts, confer a health benefit on the host”^10^. Lactobacilli represent a large and diverse group of bacteria, including many species and strains with probiotic effects. Lactobacilli have been shown to promote immune homeostasis through several mechanisms including pathogen inhibition^11–13^, intestinal barrier fortification^14,15^ and modulation of immune cell composition and function^16–23^. Much research has been invested in identifying the *Lactobacillus* (*L*.)-derived components responsible for the aforementioned effects^24,25^. Indeed, isolated peptidoglycan (PGN) from *L. salivarius* Ls33 protects mice from colitis by activation of the intracellular NOD2 receptor pathway^26^. Cell wall associated polysaccharides contribute to the ability of lactobacilli to counteract lipopolysaccharide (LPS)-induced activation of murine macrophages^27^. *Lactobacillus* and its isolated RNA directly suppress CD4^+^ T cell proliferation in a MyD88-dependent mechanism^28^, while specific DNA motifs suppress IgE-responses through activation of regulatory dendritic cells (DC)^29,30^. The secreted protein p40 promotes intestinal homeostasis by engagement of the epidermal growth factor receptor (EGFR) expressed on colonic epithelium as well as promoting IgA production via a proliferation-inducing ligand (APRIL)^31,32^. Interestingly, the highly abundant surface layer protein A (SlpA) from *Lactobacillus*, was found to supress NF-κB activation in intestinal cells while at the same time promoting inflammatory tumour necrosis factor (TNF) expression in macrophages^33^, highlighting the complexity of the cross-talk between probiotic bacteria and the host. Short-chain fatty acids (SCFA) such as butyrate and propionate (but not acetate) were found to increase *de-novo* generation of peripheral T_reg_ cells in an *in vivo* mouse model^34^. Moreover, lactic acid has a suppressive effect on IFN-γ production in human T- and NK cells^17^, and its production is a major contributor to the protective effects of lactobacilli against bacterial vaginosis^35^.

T- and NK cell activity is strongly influenced by antigen presenting cells (APC) such as DC, macrophages and monocytes. APC control lymphocyte activation through expression of co-receptors, release of chemokines and cytokines to ensure optimal pathogen clearance and to avoid tissue damage. APC-derived cytokines such as TNF, IL-1α/β, IL-6 and IL-12 enhance lymphocyte effector functions while TGF-β, IL-1 receptor antagonist (ra) and IL-10 inhibit inflammatory responses^36^. Lactobacilli are known to induce the production of innate-derived cytokines via both cell wall-derived components and secreted metabolites. Interestingly, viable lactobacilli induced IL-1β and IL-12 gene transcription in macrophages while the corresponding cell free supernatants (CFS) did not^37^. Lactobacilli-derived cell-surface components were shown to be the main inducers of inflammatory TNF production from PBMC cultures^38^, suggesting different immune stimulatory capacity of whole lactobacillus cells compared with the CFS. Soluble components derived from the gut microbiota effectively translocate from the gut lumen to peripheral tissues where they remain biologically active^39^, thus, studies regarding the effects of *Lactobacillus*-CFS on peripheral lymphocytes are indeed relevant. However, the vast majority of mechanistic studies are restricted to the intestinal epithelium and innate immune cells whereas the influence of, and mechanisms behind, lactobacilli-mediated modulation of peripheral lymphocyte responses are not fully understood.

Here, we aimed to investigate the mechanism behind the ability of *Lactobacillus*-CFS to dampen inflammatory cytokine production in peripheral lymphocytes and to identify the responsible *Lactobacillus*-derived components. We show that *Lactobacillus*-CFS promotes monocyte activation and in parallel dampens inflammatory cytokine release from lymphocytes. Size fractionation of the *Lactobacillus*-CFS revealed multiple factors with the capacity to dampen IFN-γ secretion in a monocyte-dependent manner. Furthermore, we successfully identified extracellular membrane vesicles (MVs) derived from the *Lactobacillus*-CFS, which recapitulated both the immune stimulatory and the IFN-γ dampening activity observed with the CFS alone, suggesting that gut bacteria-derived extracellular MVs are important modulators of human immunity with new implications for probiotic design.

## Material and Methods

### Ethical statement and isolation of peripheral blood mononuclear cells

Healthy, anonymous, adult volunteers (age 18–65) were included in this study, which was approved by the Regional Ethic’s Committee at the Karolinska Institute, Stockholm, Sweden {Dnr 04-106/1 and 2014/2052-32} and the methods were carried out in accordance with the approved guidelines. All study subjects gave their informed written consent. Venous blood was diluted with RPMI-1640 cell culture medium supplemented with 20 mM HEPES (HyClone Laboratories, Inc.). Peripheral blood mononuclear cells (PBMC) were then isolated by Ficoll-Hypaque (GE Healthcare Bio-Sciences AB) gradient separation. The PBMC were washed in RPMI-1640, resuspended in freezing medium containing RPMI-1640 40%, Fetal calf serum (FCS) 50% and DMSO 10%, gradually frozen in a freezing container (Mr Frosty, Nalgene Cryo 1 °C; Nalge Co.) and stored in liquid nitrogen.

### Bacterial strains and generation of cell free supernatants

*Lactobacillus rhamnosus* GG (ATCC 53103), *Lactobacillus reuteri* DSM 17938^40^ (a kind gift from BioGaia AB, Stockholm, Sweden) and *Staphylococcus aureus 161:2* (a kind gift from Åsa Rosengren, The National Food Agency, Uppsala, Sweden)^41^ were used in this study. The lactobacilli were first grown for 24 h on Rogosa agar plates (Oxoid) from which a single colony was inoculated into De Man, Rogosa and Sharpe (MRS) medium and grown as still culture overnight. The bacteria were then pelleted by centrifugation, resuspended in RPMI-1640 supplemented with glucose 18 g/l and FCS 20% at OD_600_ of 0.2 and grown as still cultures for 48 h at 37 °C, 5% CO_2_. Finally, the bacteria were pelleted by centrifugation and the CFS was pH-neutralized using 5 M NaOH, 0.2 μm filtered and stored as aliquots at −20 °C. *S. aureus* was first grown for 24 h on brain, heart infused (BHI) agar plates from which a single colony was inoculated into BHI broth and grown as still cultures for 72 h at 37 °C, 5% CO_2_. The CFS was collected by centrifugation and 0.2 μm filtered aliquots were stored at −20 °C.

### Size fractionation of the *Lactobacillus*-CFS

Size fractionation of the *Lactobacillus*-CFS was done using Amicon Ultra-4 centrifugal filter units (Merck Millipore). In brief, CFS was centrifuged for 40 min at 3400 × *g* at 4 °C in sequential order starting with the highest molecular weight cut-off (MwCO) of 100 kDa. After centrifugation, the concentrated top-fraction was re-diluted to its starting volume with RPMI-1640 cell culture medium, aliquoted and stored at −20 °C until used in assays. High performance Liquid Chromatography (HPLC) was performed using the Agilent 1260 infinity system. A Superdex 200 10/300 gel-filtration column (GE Healthcare) was equilibrated sequentially with sterile filtered water, 30% (v/v) ethanol, sterile water and finally with sterile phosphate-buffered saline (PBS), pH7.4 (0.137 M NaCl, 0.0027 M KCl, 0.01 M Na_2_HPO_4_ and 0.0018 M KH_2_PO_4_). 500 µL of *Lactobacillus*-CFS was injected into the column of the system operating at a flow rate of 0.3 mL/min and dispenser was set at 1 min slide time. Fractions were collected into a sterile 96-well plate. Consecutive fractions of 3 were subsequently pooled, aliquoted and stored at −20 °C.

### *In Vitro* stimulation of PBMC

PBMC were thawed, washed and stained with Trypan blue followed by live cell counting using a 40x light microscope. Cells were resuspended in cell culture medium containing RPMI-1640 supplemented with HEPES (20 mM), penicillin (100 U/ml), streptomycin (100 μg/ml), L-glutamine (2 mM) (all from HyClone Laboratories, Inc.) and FCS 10% (Gibco by Life Technologies) at a final concentration of 1 × 10^6^ cells/ml. Cells were seeded in flat-bottomed cell culture plates and incubated at 37 °C with 5% CO_2_ atmosphere. *S. aureus*-CFS was used as stimuli at 2.5% (v/v), lactobacilli-CFS was used at 10% (v/v) and Dynabeads human T-activator CD3/CD28 (Life Technologies) was used at 2:1 (cell:bead) ratio.

### Cell enrichment and depletion

Enriched T cells were generated from PBMC through negative selection using EasySep^TM^ Human T cell enrichment kit. To generate monocyte-depleted PBMC or B cell-depleted PBMC we used positive selection with EasySep^TM^ Human CD14 Positive Selection Kit II or EasySep^TM^ Human CD19 Positive Selection Kit II, respectively (all from STEMCELL Technologies Inc.). In brief, frozen PBMC were thawed, washed and resuspended in PBS containing FCS 2% and EDTA 1 mM at 50 × 10^6^ cells/ml. PBMC were incubated with an antibody cocktail, followed by magnetic particles and target cells were depleted or recovered using an EasySep magnet according to manufacturer’s instructions. Cell purity after depletion or enrichment was confirmed by flow cytometric analyses.

### Generation of monocyte-conditioned medium

Isolated monocytes were seeded in a 48-well culture plate at 1 × 10^6^ cells/ml and treated for 6 h with 10% LGG-CFS. Next, the monocytes were washed twice with cell culture medium and incubated in fresh cell culture medium for 14 h at 37 °C with 5% CO_2_. Finally, the monocyte-conditioned medium was collected by centrifugation and stored at 4 °C until later use.

### Proteome profiler array

The monocyte-conditioned medium was analysed for 105 secreted proteins using the Human XL Cytokine Array Kit (R&D Systems) according to manufacturer’s instructions. In brief, the stamped identification number on each membrane was cut off and replaced with an individual mark using a pencil. Each membrane was then blocked, washed and incubated overnight at 4 °C on a rocking platform with 1.5 ml of monocyte-conditioned medium diluted 1:3 using the supplied array buffer. Membranes were washed and incubated with the detection antibody cocktail for 1 h at room temperature (RT) on a rocking platform followed by addition of IRDye 800 CW Streptavidin (LI-COR Biosciences) for 30 min. Finally, the membranes were scanned and images acquired using the LI-COR Odyssey Infrared Imaging System (LI-COR Biosciences).

### Lipid removal assay

The commercial kit Cleanascite lipid adsorption and clarification reagent (Biotech Support Group) was used to remove lipids and extracellular membrane vesicles from the *Lactobacillus*-CFS according to manufacturer’s instructions. In brief, one part of Cleanascite reagent was added to four parts of *Lactobacillus*-CFS and incubated at RT for 40 min under periodical mixing. Finally, the Cleanascite reagent was separated from the CFS by centrifugation at 15000 × *g* for 1 min after which the supernatant was collected and subjected to fractionation using an Amicon Ultra-4 centrifugal filter unit (Merck Millipore) with a MwCO of 100 kDa.

### Proteinase K digestion and heat treatment

The *Lactobacillus*-CFS was subjected to protein digestion using Proteinase K immobilized to agarose (Sigma-Aldrich) using a modified protocol of that described elsewhere^42^. Proteinase K-agarose was added to *Lactobacillus*-CFS to a working concentration of 1 mg/ml and incubated with rotation at 37 °C for 1 h. Next, the Proteinase K-agarose was removed by centrifugation at 15000 × *g* for 1 min. Finally, the proteinase K digested CFS was fractionated using an Amicon Ultra-4 centrifugal filter unit (Merck Millipore) with a MwCO of 100 kDa. Fractionated control-CFS was also subjected to heat treatment at 96 °C for 15 min prior to cell stimulations.

### Isolation of extracellular membrane vesicles (MVs)

*Lactobacillus reuteri* DSM 17938 bacterial cells were grown in MRS broth (Oxoid) for 24 h at 37 °C. The bacterial cells were removed from the culture broth by centrifugation at 5,000 × *g* for 10 min at 4 °C and followed by another centrifugation at 10,000 × *g* for 10 min at 4 °C. Then the supernatants were filtrated using 0.45 μm pore filter (Millipore). Cell free supernatants were concentrated using Amicon Ultra filter unit with a MwCO of 100 kDa (which remove proteins and other molecules under 100 kDa). The supernatants were loaded on top of 12% sucrose cushion with 50 mM Tris buffer pH 7,2, with the volume ratio 5:1, and centrifuged by Beckman coulter Optima L – 80XP ultracentrifuge (Beckman coulter) at 118,000 × *g* at 4 °C for 3 h. The supernatants were discarded and the pellet was resuspended in PBS buffer and ultra-centrifuged for the second time (118,000 × *g* at 4 °C for 3 h). The pellets were then dissolved in PBS, aliquoted and stored at −70 °C.

### Nanoparticle tracking analysis (NTA)

The physicochemical characterization of MVs was also investigated by using the Nanoparticle tracking analysis (NTA). Extracellular vesicles were diluted with PBS, and directly tracked using the NanoSight NS300 system (NanoSightTM technology). The analysis was carried out according to the following instrumental set up: (i) a laser beam of 488 nm (blue); (ii) and a high-sensitivity sCMOS camera. Videos were collected and analysed using the NTA software (version 3.2), capturing a video file of the particles moving under Brownian motion. The software tracks many particles individually and using the Stokes–Einstein equation calculates their hydrodynamic diameters. Multiple videos of 90 s duration were recorded generating replicate histograms that were averaged for each sample.

### ELISA

Secreted levels of the cytokines IL-1ra (R&D Systems-BioTechne), IL-1β, IL-6, IL-10, IL-17A and IFN-γ (MabTech AB) were measured in cell culture supernatants using sandwich ELISA kits according to the manufacturer’s instructions. Absorbance was measured at a wavelength of 405 nm using a micro-plate reader (Molecular Devices Corp.) and results analysed using SoftMax Pro 5.2 rev C (Molecular Devices Corp.).

### Flow cytometry

Stimulated cells were treated with GolgiPlug/Brefeldin A for the last 4 h of incubation to block secretion of cytokines. Cells were collected and transferred to a V-shaped 96-well staining plate, stained with the LIVE/DEAD Fixable Dead Cell Stain Kit-Aqua (Life Technologies) and Fc-receptors were blocked with 10% human serum. Next, extracellular surface markers were stained with anti-CD3-PECy7 (clone: SK7), CD4-PE (clone: RPA-T4) (both from BioLegend), CD8-APCH7 (clone: SK1), CD56-APC (clone: B159) (both from BD Biosciences) and pan-γδ TCR-FITC (clone: IMMU510) (Beckman Coulter). Cells were fixed/permeabilized with the Transcription Factor buffer set (BD Biosciences) followed by intracellular staining with IFN-γ-PerCPCy5.5 (clone: B27) (BD Biosciences). Stained cells were acquired using a FACSVerse instrument with the FACS Suite software (BD Biosciences). Lymphocytes were gated based on forward/side scatter properties. Viable lymphocytes were further gated on cell surface markers; NK cells were classified as CD3^−^CD56^+^, T helper cells as CD3^+^CD4^+^, T cytotoxic cells as CD3^+^CD8^+^ and γδ T cells as CD3^+^γδ TCR^+^. Data was analysed using FlowJo Software (TreeStar).

### Statistics

**Figure 1:**
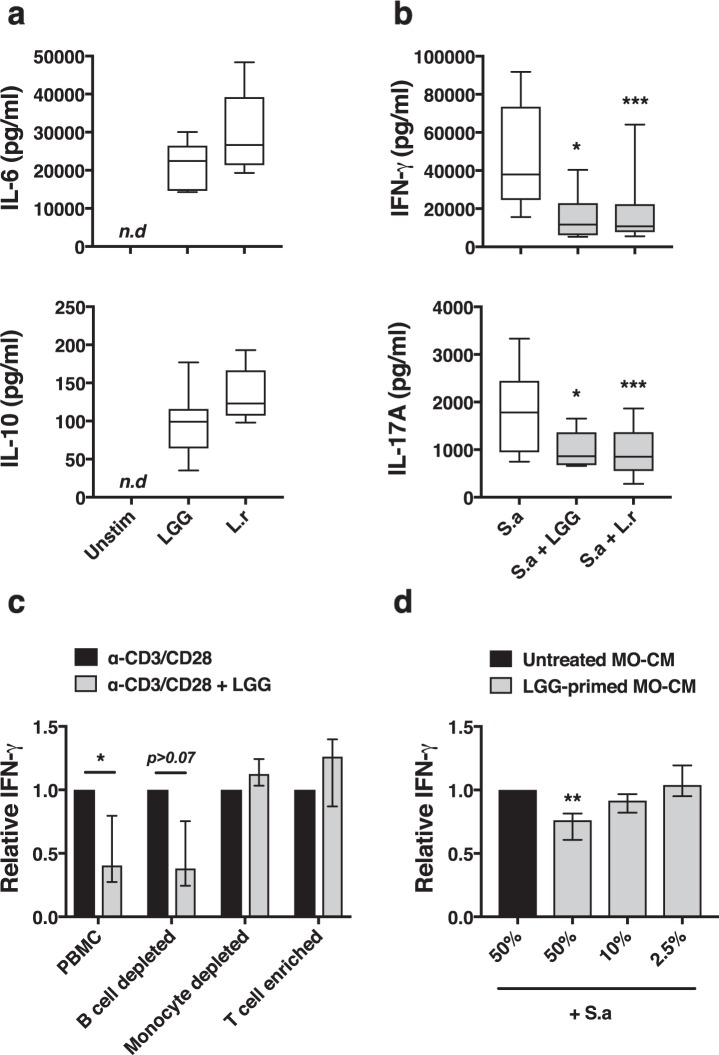
Lactobacilli-CFS dampens IFN-γ in a monocyte-dependent manner. PBMC were stimulated as indicated and culture supernatants were collected after 48 h and secreted cytokines evaluated with ELISA. (**a**) Secretion of IL-6 and IL-10 following stimulation with *LGG- or L. reuteri (L.r)*-CFS (n = 5–6). (**b**) Secretion of IL-17A and IFN-γ following stimulation with *S. aureus* (S.a)-CFS alone and in combination with *Lactobacillus*-CFS (n = 6–14). (**c**) Whole PBMC, B cell depleted, monocyte depleted or enriched T cells were stimulated with T cell specific activator beads towards CD3/CD28 alone and in combination with LGG-CFS. Secreted levels of IFN-γ was determined and normalized to stimulated cells in the absence of LGG-CFS, (n = 4–8). (**d**) Monocyte-conditioned medium (MO-CM) from isolated monocytes primed with LGG-CFS for 6 h, extensively washed and incubated in fresh cell culture medium for 14 h, was mixed 1:1 (50%), 1:10 (10%) or 1:40 (2.5%) with S.a-CFS-stimulated autologous PBMC cultures. Secreted levels of IFN-γ was quantified and normalized to stimulated cells mixed 1:1 with unprimed monocyte conditioned medium, (n = 6–8). Boxes cover data between the 25th and the 75th percentile with medians as the central line and whiskers showing min-to-max. Bar plots show median with interquartile range.

**Figure 2:**
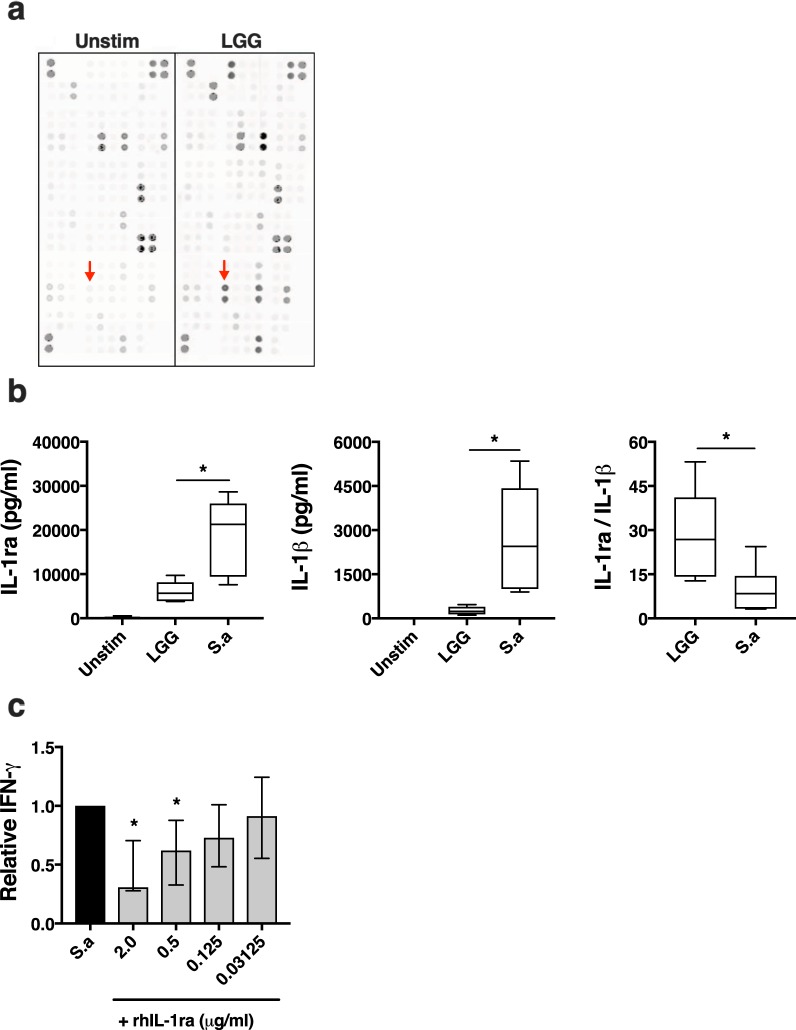
LGG-CFS induces IL-1ra from isolated monocyte cultures. Evaluation of the involvement of IL-1ra in suppression of IFN-γ production. (**a**) A membrane based cytokine array of the LGG-CFS primed monocyte-conditioned medium compared with unstimulated monocytes. Duplicate spots are positioned vertically and red arrows indicate IL-1ra. Shown is one representative membrane from two separate experiments. (**b**) Quantification of IL-1ra secretion (left), IL-1β secretion (middle) and the ratio of IL-1ra over IL-1β secretion (right) from PBMC cultures, (n = 6). (**c**) Quantification of IFN-γ secretion from *S. aureus* (S.a)-CFS stimulated PBMC in the presence of recombinant human (rh) IL-1ra. Box plots show the 25^th^ and the 75^th^ percentile with median value as the central line and whiskers show min-to-max. Bar plot shows median with interquartile range.

**Figure 3:**
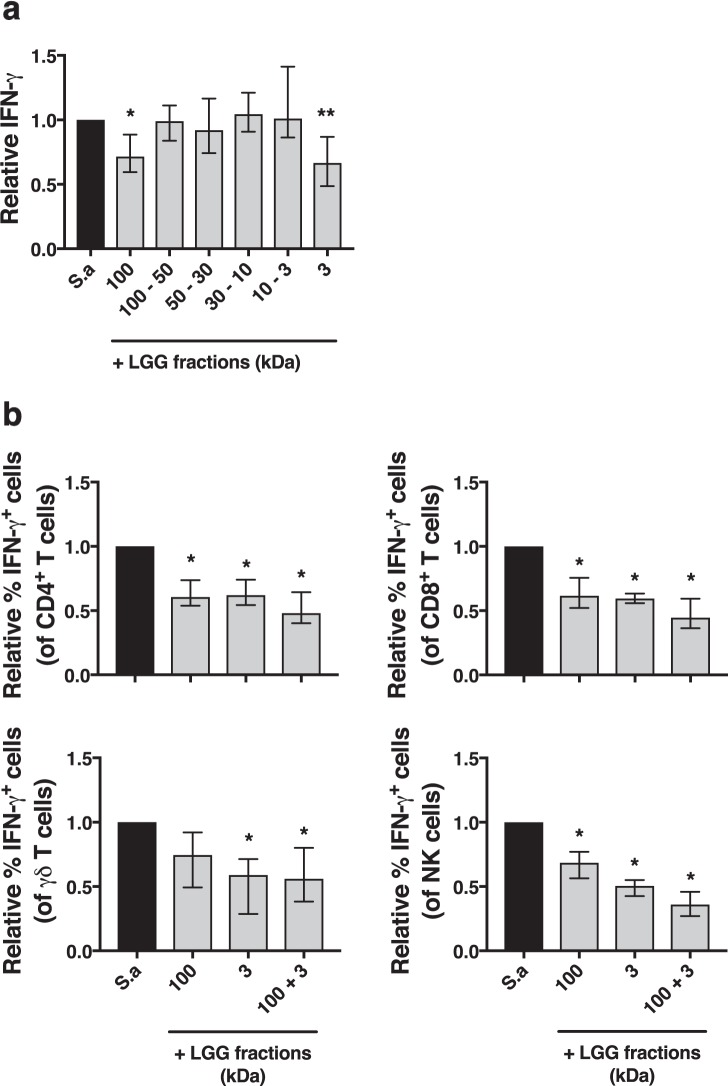
Size fractionation of lactobacilli-CFS reveals multiple factors with IFN-γ dampening activity. Spin-column fractionation was employed to generate fractions of the LGG-CFS ranging from larger than 100 kDa to smaller than 3 kDa in molecular weight, whereby the IFN-γ dampening capacity of each fraction was evaluated on PBMC stimulated 48 h with *S. aureus* (S.a)-CFS. (**a**) Quantification of secreted levels of IFN-γ from stimulated PBMC normalized to S.a-CFS alone, (n = 8). (**b**) Flow cytometric analyses of intracellular IFN-γ expression in CD4^+^ T cells, CD8^+^ T cells, γδ T cells and NK cells normalized to S.a-CFS alone, (n = 6). Shown are medians with interquartile range.

**Figure 4:**
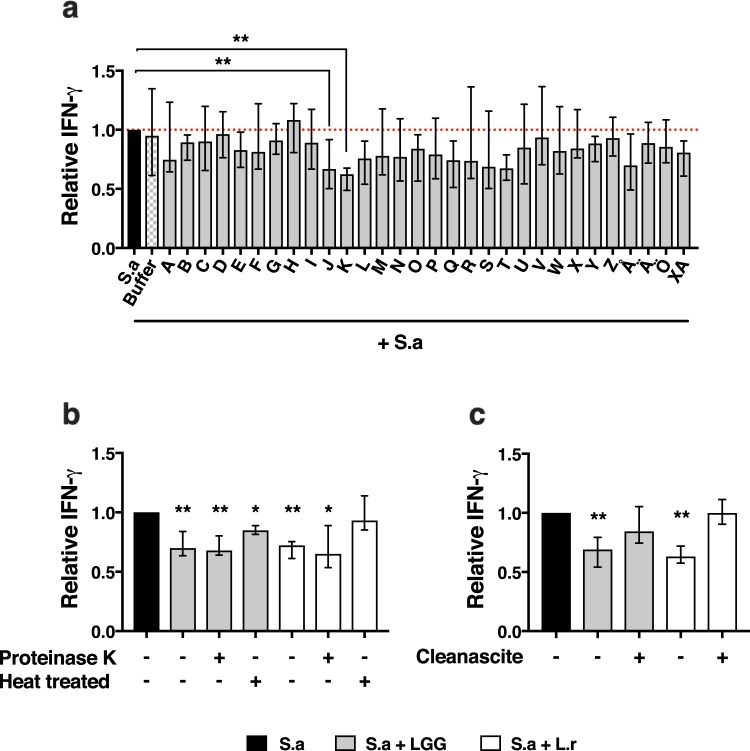
The lactobacilli-CFS high Mw fraction contains an isolated heat sensitive lipid-based factor. Molecular characterization of the IFN-γ dampening factor of the *Lactobacillus*-CFS high Mw fraction. The *Lactobacillus*-CFS high Mw fraction was subjected to high performance liquid chromatography (HPLC), proteinase K treatment, heat inactivation or delipidation through lipid adsorption before being added to *S. aureus* (S.a)-CFS stimulated PBMC. (**a**) The LGG-CFS was spin-column fractionated with a pore-size of 100 kDa, after which the top fraction was collected and subjected to further size fractionation using HPLC. The HPLC generated fractions were added to S.a-CFS stimulated PBMC for 48 h followed by quantification of secreted levels of IFN-γ normalized to S.a-CFS alone, (n = 8). (**b**) PBMC were stimulated 48 h in the presence or absence of proteinase K treated, heat treated and control *Lactobacillus*-CFS whereby relative secreted levels of IFN-γ was analysed, (n = 8). (**c**) PBMC were stimulated 48 h in the presence or absence of delipidated or control *Lactobacillus*-CFS whereby relative secreted levels of IFN-γ was analysed (n = 8). Shown are medians with interquartile range.

**Figure 5:**
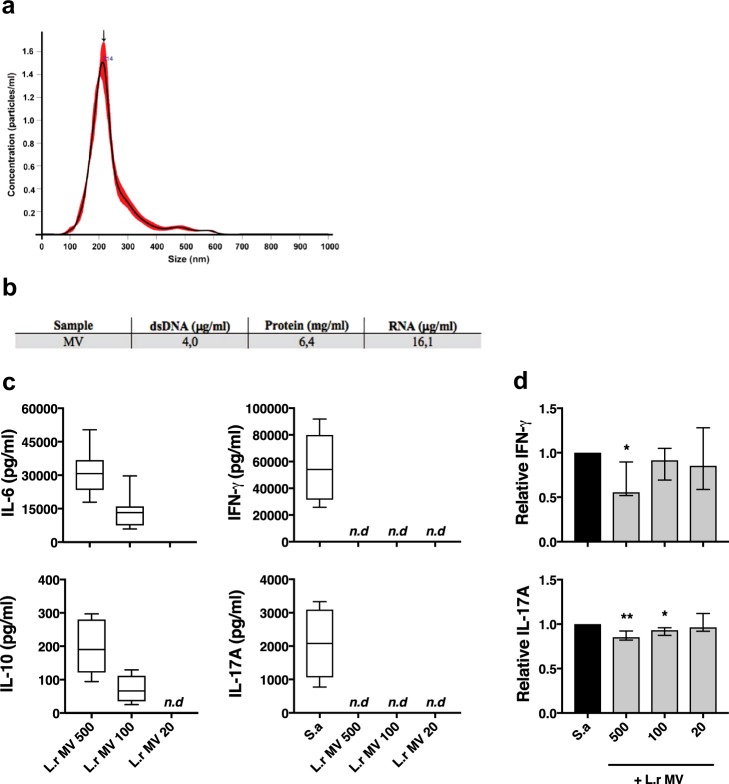
Lactobacilli-derived extracellular membrane vesicles are immune stimulatory and dampen IFN-γ and IL-17A responses. Characterization and evaluation of the immunomodulatory effects of purified *L. reuteri*-derived extracellular membrane vesicles (MVs) in PBMC cultures. (**a**) The nanoparticle tracking distribution of isolated MVs from *L. reuteri* is representative of three independent replicates. The size distribution represents the wide distribution of vesicles with the arrow marking the mean peaks of particles at 214 nm and a concentration of 1,6 × 10^12^ particles/ml. (**b**) Quantification of dsDNA, protein and RNA content of the MVs isolates. (**c**) PBMC were cultured for 48 h in the presence of *L. reuteri* (L.r)-MVs at 500:1, 100:1, and 20:1 (MV:cell) ratio followed by quantification of secreted levels of IL-6, IL-10, IL-17A and IFN-γ (n = 8). (**d**) PBCM were stimulated with *S. aureus* (S.a)-CFS (2.5%) in the presence of L.r-MVs at 500:1, 100:1 and 20:1 (MV:cell) ratio followed by quantification of secreted levels of IFN-γ and IL-17A. Shown are relative values normalized to *S. aureus*-CFS alone, (n = 8). Boxes cover data between the 25th and the 75th percentile with medians as the central line and whiskers showing min-to-max. Bar plots show median with interquartile range.

All statistical tests were done using GraphPad Prism (GraphPad Software). All data was considered non-parametric. For Figs. 1–5, the Wilcoxon test was employed. The Friedman test followed by Dunn’s multiple comparisons was employed in Fig. 4a. Differences were considered significant when p < 0.05 and the following significance levels were used *P < 0.05; **P < 0.01; ***P < 0.001.

## Results

### Lactobacilli dampen IFN-γ responses by induction of a monocyte-derived soluble inhibitor

We have previously shown that cell free supernatants (CFS) from lactobacilli grown in their regular MRS medium dampen IFN-γ and IL-17A responses from T- and NK cells^17,20^. In the present study we cultivated the lactobacilli in modified cell culture medium, a more physiologically relevant medium for immune cells. PBMC cultured in the presence of CFS from *L. rhamnosus* GG (LGG) or *L. reuteri* DSM 17938 secreted IL-6 and IL-10 (Fig. 1a), showing that lactobacilli promote secretion of innate cytokines when grown in cell culture medium. Furthermore, both LGG- and *L. reuteri*-CFS significantly dampened *S. aureus*-induced IFN-γ and IL-17A secretion (Fig. 1b), suggesting that lactobacilli have the capacity to promote innate responses while suppressing adaptive inflammatory responses.

Activation of T- and NK cells is regulated by innate APC such as monocytes^43,44^. We therefore investigated whether the lactobacilli-mediated dampening of IFN-γ requires the presence of monocytes or B cells. We depleted PBMC of either monocytes or B cells or isolated pure T cells, prior to stimulation with a T cells specific activator towards CD3/CD28 alone or in combination with LGG-CFS. Depletion of B cells did not affect the IFN-γ-dampening activity while depletion of monocytes completely abolished the LGG-CFS-mediated IFN-γ suppression. In accordance with this, the LGG-CFS was not able to dampen IFN-γ secretion in pure T cell cultures (Fig. 1c). Next, we sought to investigate if the monocyte-dependent dampening of IFN-γ required physical contact between the monocyte and the IFN-γ-producing cell. We isolated and primed monocytes for 6 h with LGG-CFS, thoroughly washed and incubated them in fresh cell culture medium for an additional 14 h. The conditioned medium from the LGG-primed monocytes was subsequently added to stimulated autologous PBMC cultures. We observed a significant reduction in IFN-γ secretion by the monocyte-conditioned medium in a dose-dependent manner (Fig. 1d), suggesting that LGG dampens IFN-γ responses by inducing a monocyte-derived soluble inhibitor.

### *Lactobacillus*-primed monocytes secrete IL-1ra

In order to identify the IFN-γ suppressive monocyte-derived factor, the monocyte-conditioned medium was analysed on a proteomic array. Only twelve of the measured factors were increased in LGG-treated samples compared with unstimulated conditions (Table 1), of which IL-1ra was the candidate molecule that was most likely to exert immune regulatory effects on T cells (Fig. 2a). IL-1ra is an antagonistic inhibitor of the pro-inflammatory IL-1 signalling pathway and it has been shown to reduce IFN-γ secretion^45^. We observed significantly higher levels of IL-1ra in response to *S. aureus* alone compared to LGG, however, *S. aureus* also induced significantly more IL-1β. In fact, we found that LGG alone induced a 3-fold higher ratio of IL-1ra/IL-1β than *S. aureus* alone (Fig. 2b), indicating that LGG favours an anti-inflammatory IL-1 signalling pathway. Finally, supplementation of recombinant IL-1ra to *S. aureus* stimulated PBMC resulted in reduced IFN-γ levels in a dose-dependent manner (Fig. 2c). Collectively, these data indicate that lactobacilli promote the anti-inflammatory IL-1ra signalling pathway and that this may contribute to their IFN-γ-dampening effect.

**Table 1:** Proteome Profiler Array results of supernatants obtained from LGG-treated or unstimulated isolated human monocytes.

*Protein*	LGG-treated monocytes	Unstimulated monocytes
	*Increased as compared to unstimulated*	*Detected*
CXCL5	Yes	Yes
CCL2	Yes	Yes
uPAR	Yes	Yes
MMP-9	Yes	Yes
CXCL1	Yes	No
CCL7	Yes	No
CCL20	Yes	No
IL-1ra	Yes	No
IL-6	Yes	No
IL-10	Yes	No
Angiopoietin-2	Yes	No
MIP-1α/β	Yes	No

### Lactobacilli dampen IFN-γ in T cells and NK cells through multiple factors of different molecular size

Several components of lactobacilli have been shown to be important for host cell interactions, in particular proteins, nucleic acids, PGN and SCFA^26,46,47^. In order to isolate the factors that mediate the IFN-γ dampening activity, we subjected the lactobacilli-CFS to size exclusion fractionation generating multiple fractions spanning from less than 3 kDa to larger than 100 kDa in molecular weight (Mw) and stimulated PBMC in the presence of each isolated fraction and measured secretion of IFN-γ. Two fractions of the LGG-CFS, the 3 kDa (from hereinafter referred to as “low Mw fraction”) and the 100 kDa (from hereinafter referred to as “high Mw fraction”), were able to dampen *S. aureus*-induced IFN-γ production (Fig. 3a). Interestingly, the dampening activity of the *L. reuteri*-CFS was also confined within the same two fractions of the CFS as for LGG (data not shown), suggesting that the mechanism of cytokine modulation is conserved between both species. Finally, we analysed IFN-γ expression on a cellular level using flow cytometry and found that both the high and the low Mw fractions significantly reduced the frequency of IFN-γ expressing cells within the CD4^+^ and CD8^+^ T cell, γδ T cell and NK cell populations (Fig. 3b).

### The lactobacilli-CFS high Mw fractions contain a heat sensitive lipid-based factor

During size fractionation, lactobacilli-derived metabolites and small compounds end up in the low Mw fraction of the CFS. We here focused our attention on the cytokine modulating capacity of the high Mw fraction. In order to increase the resolution of the fractionation, the high Mw fraction was further fractionated using high performance liquid chromatography (HPLC). An additional 30 fractions were generated and tested for IFN-γ dampening activity on *S. aureus*- stimulated PBMC. We identified two consecutive fractions, named J and K, which consistently dampened IFN-γ secretion (Fig. 4a). Furthermore, comparing the protein absorption chromatogram with the ability to dampen IFN-γ, fractions J and K was found to completely match the first protein peak to be eluted from the HPLC column, which indeed was absent in the growth medium control (See Supplementary Fig. S1). Importantly, the protein absorption chromatogram from LGG and *L. reuteri* looked near identical, further supporting that the IFN-γ-dampening elicited by both species involved the same factor and mechanism. In order to further characterize the isolated factor, we subjected the lactobacilli-CFS to protein digestion using agarose-immobilized proteinase K followed by size fractionation with a 100 kDa filter and assessed the IFN-γ dampening activity of the proteinase K treated high Mw fraction on *S. aureus*-stimulated PBMC. Interestingly, no difference in the ability of LGG or *L. reuteri* to dampen IFN-γ was observed after proteinase K treatment. Heat inactivation of the *L. reuteri*-CFS, but not LGG-CFS, prior to stimulations abolished the mediated IFN-γ dampening (Fig. 4b). The lactobacilli-CFS was treated with a lipid adsorption matrix to remove all lipids, including extracellular membrane vesicles (MVs) that are enclosed by a lipid membrane, before being added to stimulated PBMC. Indeed, the IFN-γ dampening was lost for both LGG and *L. reuteri* after lipid removal (Fig. 4c).

### Lactobacilli-derived extracellular MVs dampen IFN-γ and IL-17A secretion

Lactobacilli are known to produce MVs^48^ and these bacterial MVs are also know to contain membrane integrated proteins as well as intravesicular proteins that, due to the membrane enclosure, are protected from enzymatic digestion while still susceptible to heat inactivation^49^. We isolated MVs from both species of lactobacilli and a physiochemical characterization of the *L. reuteri*-MVs revealed a size distribution ranging from 100 nm to 400 nm in diameter, with an average size of 214 nm (Fig. 5a) and contained proteins, DNA and RNA (Fig. 5b). In order to test whether the MVs are responsible for the cytokine-modulatory effect observed with the lactobacilli-high Mw fraction, isolated MVs were added to PBMC at a MV-to-cell ratio of 500:1, 100:1 and 20:1 and incubated for 48 h. The cell culture supernatants were collected and analysed for induction of cytokines using ELISA. The *L. reuteri*-MVs clearly induced the production of both IL-6 and IL-10 in a concentration dependent manner, while no IFN-γ or IL-17A was detected (Fig. 5c). Moreover, adding isolated MVs to *S. aureus*-stimulated PBMC significantly dampened IFN-γ and IL-17A secretion to a similar extent as the CFS-high Mw fraction (Fig. 5d). Again, a substantial dampening of IFN-γ and IL-17A was also observed using LGG-derived MVs (data not shown).

## Discussion

In the current study, we aimed to explore the underlying mechanism behind the cytokine-modulatory effects of lactobacilli. Here we show that lactobacilli produce soluble factors that induce the production and secretion of chemokines (Table 1) and cytokines such as IL-1β, IL-6 and IL-10 from PBMC cultures. We identify extracellular MVs as being one of the main contributors to both the immune-stimulatory and immune-dampening activity of the lactobacilli-CFS, possibly by increasing the relative abundance of IL-1ra to IL-1β by a monocyte-dependent mechanism.

Lactobacilli and its components have been shown to influence T cell differentiation and proliferation in a T cell intrinsic manner. *Lactobacillus*-derived RNA suppress CD4^+^ T cell proliferation in the absence of APC^28^ and SCFA increase extrathymic generation of T_reg_ cells by promoting acetylation of the *foxp3* gene locus within the naïve T cell population itself^34^. In the current study, we also investigated whether the lactobacilli-CFS-mediated dampening of IFN-γ was dependent solely on T cell intrinsic factors or not. Interestingly, IFN-γ dampening was fully dependent on the presence of monocytes since the *Lactobacillus*-CFS had no effect on IFN-γ production from isolated T cell or monocyte-depleted PBMC cultures (Fig. 1b). Furthermore, we observed a significant level of dampening by using *Lactobacillus*-primed monocyte-conditioned medium alone, confirming that the dampening mechanism is cell-to-cell contact independent.

Innate cells are known to regulate lymphocyte activation through soluble mediators^50^. IL-10 is a regulatory cytokine with an important role in preventing excessive inflammation by increasing T_reg_ cell function and reducing inflammatory cytokine responses. Lactobacilli-induced IL-10 is described as an important mechanism underlying the beneficial effects of probiotic bacteria on excessive immune activation^51,52^. However, neutralizing IL-10 does not affect the ability of lactobacilli-CFS to dampen IFN-γ production from stimulated PBMC^17^. Moreover, studies in IL-10 KO mice models show that lactobacilli can still modulate immune responses and ameliorate intestinal inflammation by an IL-10-independent mechanism^53,54^, indicating multiple mechanisms of action involving lactobacilli-mediated immune modulation. IL-1ra is another anti-inflammatory cytokine that suppress immune activation by antagonistic competition with IL-1α/β signalling. IL-1 signalling is important for both T- and NK cells effector responses, in particular for IL-17A and IFN-γ production, respectively^55,56^. IL-1β has also been identified as a driver of inflammation in (certain) intestinal inflammatory diseases^57^ and neutralizing IL-1β using IL-1ra inhibits fungal-induced IFN-γ responses^45^. In the current study we confirmed IL-1ra production from PBMC cultured with either *Lactobacillus*-CFS or *S. aureus*-CFS. However, *Lactobacillus*-CFS induced minimal levels of IL-1β (Fig. 2b), while *S. aureus* induced significantly larger amounts of IL-1β. In fact, *Lactobacillus*-CFS treatment resulted in a 3-fold higher ratio of IL-1ra/IL-1β compared with *S. aureus-*CFS. Moreover, adding recombinant IL-1ra to *S. aureus-*stimulated PBMC resulted in suppressed IFN-γ responses, supporting the involvement of IL-1 signalling in *Lactobacillus*-mediated dampening of induced IFN-γ.

In a previous study, we confirmed the IFN-γ-dampening effect of lactate, specifically on unconventional mucosal associated invariant T (MAIT) cells, γδ T cells and NK cells but not conventional T cells. Here, we showed that the low fraction of the *Lactobacillus*-CFS, containing only molecules of less than 3 kDa in molecular weight, was able to dampen IFN-γ production in all T cell subtypes including conventional CD4^+^ and CD8^+^ T cells. This indicates that lactobacilli produce low molecular weight molecules, other than lactate, capable of modulating pro-inflammatory responses in T cells. Lactobacilli produce several SCFA such as propionate, acetate and butyrate with known effects on host physiology and immunity^34^.

*Lactobacillus* is a highly heterogeneous genus with immune stimulatory activity that varies between species and strains^58,59^. Interestingly, the immune dampening activity we observed in our setting was conserved across species and strains (See Supplementary Fig. S2). The ability to produce extracellular MVs is also a generally conserved trait as it has been found among multiple bacterial genera, species and strains^60^. Treatment of the lactobacilli-CFS high Mw fraction with a lipid removal agent rendered both LGG and *L. reuteri* incapable of modulating IFN-γ production, indicating that MVs are involved for both species tested. Proteinase K treatment of the *L. reuteri*-CFS high Mw fraction had no impact on IFN-γ-dampening while heat inactivation did, suggesting that the MV-dampening involves delivery of intravesicular protein cargo to the target cells. On the other hand, heat inactivation of the LGG-CFS high Mw fraction did not abolish the dampening effect to a significant level, suggesting that there are differences in the MV-mediated modulation of cytokine responses between LGG and *L. reuteri*. Recently, bacterial MVs have been implicated in a broad range of functions in relation to bacteria-host interaction. MVs from *Lactobacillus* have been shown to inhibit hepatic cancer growth^61^, protect against *S. aureus*-induced atopic dermatitis^62^ and upregulate host immune gene transcription associated with defence against pathogenic infections^63^. The exact mechanism behind bacterial MV-associated host modulation is still not clear. MVs isolated from *Bifidobacterium longum* is internalized by mast cells in a phagocytosis-independent mechanism^64^, while MVs isolated from *L. sakei* subsp. *sakei* enhance IgA production by activation of Toll-like receptor 2 signalling^65^. Whether or not the MVs are taken up by the monocytes or interact solely with surface receptors, in our settings, remains to be investigated. Moreover, a protein analysis of purified *Lactobacillus-*derived MVs revealed the presence of several proteins (p40, p75) with known probiotic effects^66^. Indeed, the MVs, which we isolated from *L. reuteri*, did contain proteins and nucleic acids (Fig. 5b). Furthermore, gut bacteria-derived MVs have been isolated from peripheral blood, and the circulating MV-profile has been associated with neurodegenerative disease^67^, strengthening the relevance of our findings.

Collectively, we have shown that lactobacilli secrete multiple immunomodulatory factors with the capacity to counteract lymphocytic pro-inflammatory cytokine production by promoting monocyte-derived IL-1ra. Additionally, we identified *Lactobacillus*-produced extracellular MVs as a significant component for the cytokine regulatory effects observed, extending the general knowledge of bacteria-host interactions, which could bring new implications for future probiotic design.

## Supplementary information

Supplementary information

## Data Availability

The datasets generated during and/or analysed during the current study are available from the corresponding author on reasonable request.
