# Supplementary material for: Extracellular Membrane Vesicles from Lactobacilli Dampen IFN-γ Responses in a Monocyte-Dependent Manner
Source: Sci Rep. 2019 Nov 19;9:17109. doi: 10.1038/s41598-019-53576-6 (PMC6864076; doi:10.1038/s41598-019-53576-6)
Supplement: Supplementary file 1 — Supplementary information [file 41598_2019_53576_MOESM1_ESM.pdf]

Extracellular Membrane Vesicles from Lactobacilli Dampen IFN-γ Responses in a Monocyte-Dependent Manner

Manuel Mata Forsberg[1], Sophia Björkander[1*], Yanhong Pang[2*], Ludwig Lundqvist[2], Mama Ndi[3], Martin Ott[3], Irene Buesa Escribá[1], Marie-Charlotte Jaeger[1], Stefan Roos[2], Eva Sverremark-Ekström[1*]

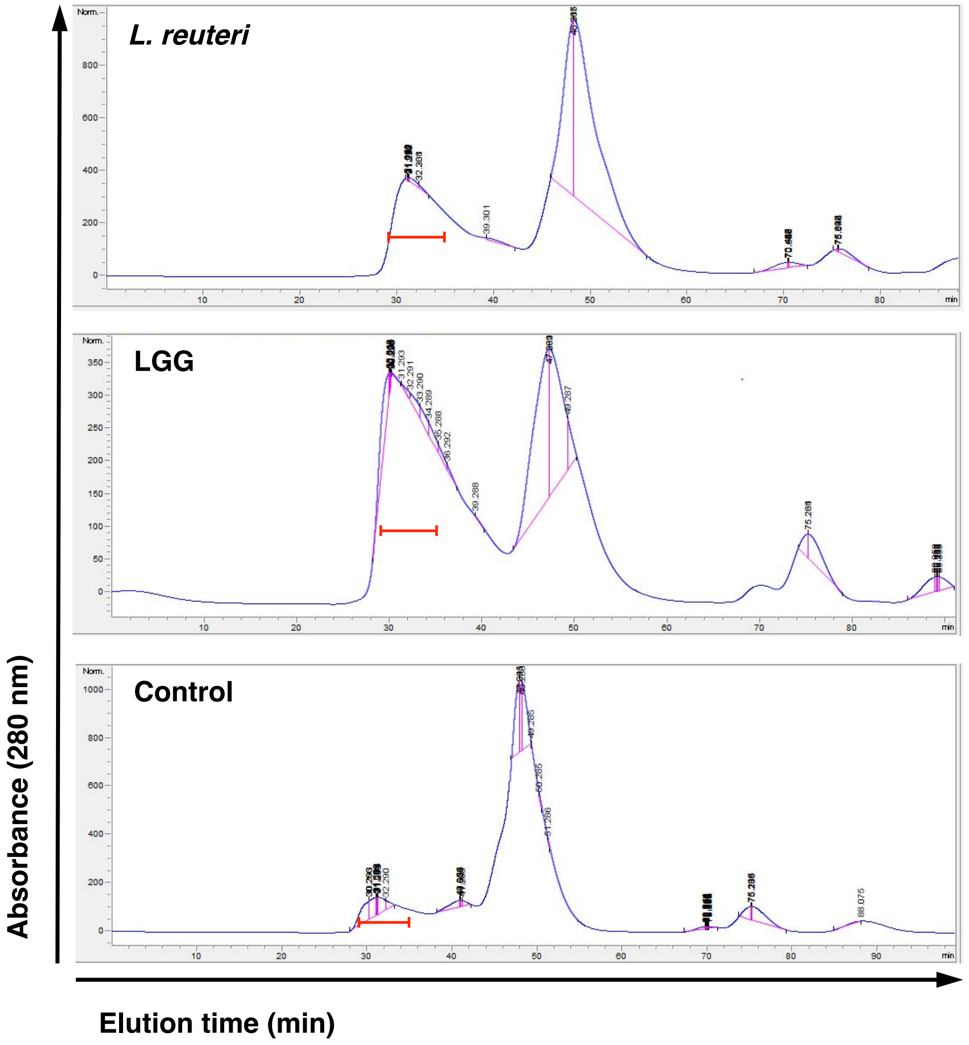

Supplementary Figure S1. Protein absorption chromatograms from HPLC fractionation of the lactobacilli-CFS high Mw fraction

Lactobacilli were grown for 48 h in modified cell culture medium, the supernatants were collected and sterile filtered. The CFS was pre-fractionated using a spin-column with a pore size of 100 kDa before being subjected to high performance liquid chromatography (HPLC) fractionation. Shown are absorbance values at 280 nm wavelength over time in minutes from *L. reuteri* (top), LGG (middle) and growth medium control (bottom) samples. Fractions within 3 minutes retention time were subsequently pooled in order and assigned to an alphabetical letter. Red bars indicate fraction J-K.

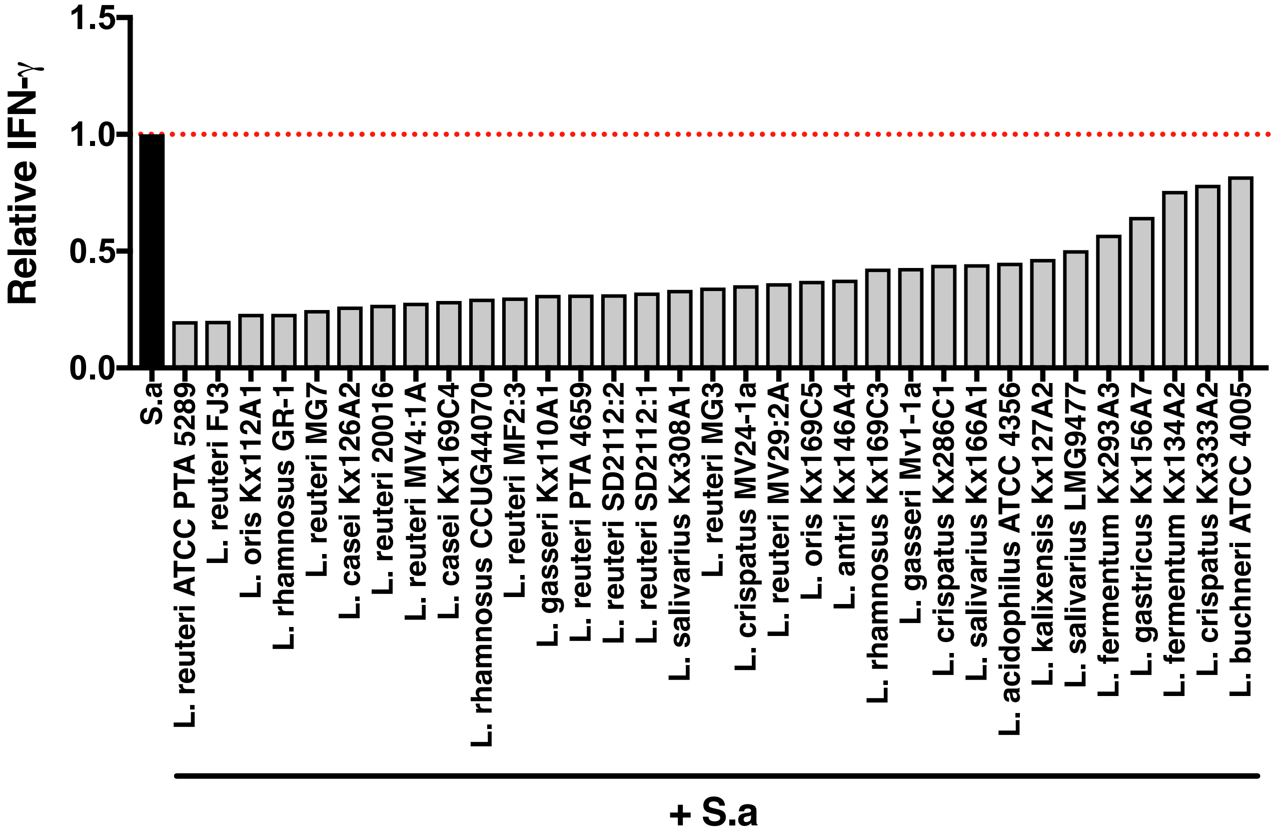

Supplementary Figure S2. IFN-γ dampening activity is conserved across multiple *Lactobacillus spp.* and strains

Evaluation of IFN-γ dampening by selected *Lactobacillus* spp. and strains. PBMC were stimulated with *S. aureus* (S.a)-CFS in the presence or absence of *Lactobacillus*-CFS for 48 h. Secreted levels of IFN-γ was quantified and normalized to *S.a*-CFS alone. Data shown is representative of one donor.
